# Bempedoic Acid, an Inhibitor of Cholesterol Biosynthesis, Reduces Cardiovascular Events

**DOI:** 10.3390/jcm12103463

**Published:** 2023-05-14

**Authors:** Ishwarlal Jialal, Samuel Olatunbosun

**Affiliations:** VA Medical Center, 10535 Hospital Way, Mather, CA 95655, USA; samuel.olatunbosun@va.gov

An elevated low-density lipoprotein cholesterol (LDL-C) is a major risk factor for premature atherosclerotic cardiovascular diseases (ASCVD). In addition to therapeutic lifestyle changes, pharmacotherapy is required to lower LDL-cholesterol. The available therapies known to reduce LDL-cholesterol and ASCVD events include 3-hydroxy-3-methylglutaryl–coenzyme A (HMG-CoA) reductase inhibitors known as statins, bile acid sequestrants, ezetimibe, and proprotein convertase subtilisin–kexin type 9 (PCSK9) inhibitors [1,2,3].

Bempedoic Acid (BA) is a first-in-class inhibitor of ATP-citrate lyase (ACL), upstream of the HMG-CoA reductase in the cholesterol biosynthetic pathway, resulting in decreases in LDL-cholesterol and apolipoprotein B [4,5,6,7,8]. It is a prodrug that is activated in the liver by long-chain Acyl CoA Synthase 1 (ACSVL1) to Bempedoyl-CoA, the active metabolite that inhibits ACL, resulting in a decrease in cholesterol biosynthesis, which culminates in an upregulation of hepatic LDL receptors, promoting a decrease in the circulating LDL-C [4,5,6,7,8].

Based on 4 Cholesterol lowering via BA, an ACL-inhibiting regimen (CLEAR) program, BA has been approved for patients with familial hypercholesterolemia and patients with ASCVD not attaining LDL-C goals despite receiving the maximum tolerated statin therapy [4,5,6,7,8]. It was well tolerated and effective in patients who were statin intolerant [5,6,7,8]. However, until recently, there was no evidence of a reduction in ASCVD events with BA. 

The CLEAR Outcomes trial was a double-blind, placebo-controlled, randomized trial on 13,970 patients, ranging in age from 18 to 85 years, who were unable or unwilling to undergo statin therapy due to unacceptable adverse reactions (statin intolerance) and had ASCVD or were at a high risk for ASCVD [9]. The patients (n = 6992) were assigned to BA (180 mg/day) or a placebo (6978). The patients were followed up for a median duration of 40.6 months. The primary end-point was the composite of four components of major adverse ASCVD events: death from cardiovascular causes, a non-fatal myocardial infarction, non-fatal stroke, and coronary revascularizations. The Outcomes trial had a 90% power in detecting a 15% reduction in these ASCVD events. The patients on low-dose statin without unacceptable side effects, ezetimibe, niacin, bile acid sequestrants, fibrates, and PCSK9 inhibitors administered as monotherapy or in combination therapy were included. Only 23% of the patients were on statin therapy. The patients had a mean age of 65.5 years, 41% of which were <65 years old and 91.5% of which were white. There was a good representation of both women (48%) and diabetic patients (45%). Seventy percent of the patients had experienced a previous ASCVD event (secondary prevention) and the remaining 30% were primary prevention patients. The mean LDL-C level was 139 mg/dL and that of the HDL-C was 49.6 mg/dL. The median triglycerides (TG) level was 159.5 mg/dL and for high-sensitivity C-reactive protein (hsCRP), it was 2.3 mg/L. It appears that 20.6% of the patients had an estimated glomerular filtration rate between 30–59 mL/min/m^2^.

At 6 months, the mean LDL-C was 107 mg/dL in the BA group compared to 136 mg/dL in the placebo group, a significant 21.7% reduction. Over the duration of the trial, the time average LDL-C reduction with BA was 15.9%. The percentage reduction in the hsCRP was 21.6% at 6 months and the lower hsCRP levels in the BA persisted throughout the duration of the trial. There appeared to be no significant effect on the TG levels (−4.8%); however, there seemed to be a significant reduction in the HDL-C levels of −5.7%.

There was a significant reduction in the composite primary end-point (death from cardiovascular causes, a non-fatal myocardial infarction, non-fatal stroke, and coronary revascularizations), with a hazards ratio (HR) of 0.87 and 95% confidence intervals (CI) of 0.79 to 0.96, *p* = 0.004. The *p* for the interaction suggests that the patients in the primary prevention group accrued a greater benefit than those of in the secondary prevention group. The key secondary end-point reductions contributing to the reduction in the primary end-point included fatal and non-fatal myocardial infarction, with a HR of 0.77 with 95% CIs of 0.66 to 0.91, *p* = 0.002; and coronary revascularizations, with a HR of 0.81 with 95% CIs of 0.72 to 0.92, *p* = 0.001. There was no significant decrease in fatal and non-fatal stroke (HR of 0.85 with 95% CIs of 0.67 to 1.07), death from cardiovascular causes (HR of 1.04 with 95% CIs of 0.88 to 1.24), and mortality from any cause (HR of 1.03 with 95% CIs of 0.90–1.18).

A patient-level analysis of the pooled data from phase 3 clinical studies showed favorable effects on the glycemic indices across all the groups (diabetes, prediabetes, and normoglycemia), which were albeit not statistically significant for each group [10]. Of note is that the patients with diabetes and prediabetes treated with BA experienced a statistically significant but modest reduction in HbA1C and weight compared to their counterparts in the placebo group. These findings were consistent with the results from some previous studies. The CLEAR Outcomes trial publication [9] did not particularly delve into the glycemic impact of BA, whereas a detailed analysis of the data from other, though smaller, studies implied a favorable effect. Based on our knowledge of the biochemical pathways involved, it is plausible that an ACL inhibitor could have multiple desirable metabolic effects that go beyond lipid control. This is because ACL is involved not only in cholesterol synthesis, but in carbohydrate and fatty acid metabolism as well [4,5]. However, there was no increase in new onset diabetes with BA in the Outcomes trial.

Furthermore, BA lowered hsCRP across all the glycemic strata. The reduction in hsCRP levels, the prototypic marker of inflammation, suggests that the concomitant reduction in both the LDL-C and hsCRP contributed to the reduction in the ASCVD events [11]. The authors did not perform such analyses, but the patients with hsCRP levels of >2.0 mg/L appeared to have accrued a greater benefit in the subgroup analyses [9].

Statin intolerance is most commonly due to muscle symptoms. Treatment with BA was not associated with skeletal muscle adverse effects in early clinical trials. This has now been validated by this robust and large multinational study, the CLEAR Outcomes trial [9]. In the clinical trial, the incidences of any reported muscle disorder adverse events were similar in both the BA and placebo groups, at 15.0% and 15.4%, respectively, and for myalgia specifically, 5.6% in the BA group versus 6.8% in the placebo group. The blood creatine phosphokinase increased in 2.3% of the patients receiving BA and 2.0% in the placebo group. Only two patients in the entire study, one in the BA group and the other in the placebo group, met the diagnostic criteria for rhabdomyolysis. The incidences of muscle disorder adverse events leading to drug discontinuation were similar in the two trial groups (2.9% in BA group versus 3.2% in the placebo group).

A few notable adverse effects were observed in the CLEAR Outcomes clinical trial. As previously reported, hyperuricemia was more common in the BA group than in the placebo group (10.9% versus 5.6%), as well as the incidence of gout (3.1% versus 2.1%). The mean increase from baseline in the creatinine levels was greater in the BA group in comparison with that in the placebo, and additionally, the occurrence of renal events, defined as renal function impairment, was higher, at 11.5% in the BA group versus 8.6% in the placebo group. The hepatic enzyme levels increased by 4.5% in the BA group, more than the 3.0% increase in the placebo group. The incidence of cholelithiasis was higher for BA, at 2.2% in the BA group versus 1.2% in the placebo group, a notable new finding, as this had not been observed in any of the previous clinical trials.

In the CLEAR Outcomes trial [9], the overall incidences of adverse events, serious adverse events, and adverse events leading to a discontinuation of the treatment regimen did not differ meaningfully between the BA group and placebo group.

In conclusion, based on our current understanding of the pathophysiology of ASCVD and the published data on the benefits and risks of BA, it can be considered a valuable addition to a clinician’s armamentarium in combating cardiovascular disease by lowering LDL-C in primary and secondary prevention, especially for statin-intolerant patients. In the Outcomes trial, it reduced the primary end-point by 13%. It is important to emphasize that the effect of BA was not tested in patients on conventional doses of statins [9] and, since it increases the drug levels of simvastatin and pravastatin, it should not be used with simvastatin in doses of >20 mg/d and pravastatin in doses of >40 mg/d [4,5]. Additionally, given the duration of the trial, there was no benefit on cardiovascular mortality.

Statin is still the gold standard for LDL-C-lowering therapy. However, BA may have a unique role for patients who cannot tolerate or are unwilling to take statins. The incidence of muscle-related adverse effects are low with BA therapy [9], as it is an oral prodrug activated by the ACSVL1 primarily in the liver and not in the muscle cells.

Another advantage of BA over statin, which was previously suspected and is now corroborated by the findings from the CLEAR outcomes trial, is the avoidance of new onset diabetes or the worsening of hyperglycemia. Additionally, BA in combination with ezetimibe is an attractive option for patients that have declined treatment with injectable non-statin therapies [4,5].

As noted above, BA has its own potential adverse effects; hence, it needs to be used with caution or avoided in certain situations based on clinical judgement. These include patients with a history of uncontrolled gout and perhaps cholelithiasis.

The cost of BA, which is currently available only as a branded product, still poses a significant challenge. The availability of a combination preparation of BA and ezetimibe may be useful for patients who require additional lowering and/or patients with adherence problems to multidrug regimens [8].

## Data Availability

The data are available from the senior author for review upon reasonable request.
